# 18β-Glycyrrhetinic-acid-mediated unfolded protein response induces autophagy and apoptosis in hepatocellular carcinoma

**DOI:** 10.1038/s41598-018-27142-5

**Published:** 2018-06-19

**Authors:** Jin Chen, Zhao-qi Zhang, Jia Song, Qiu-meng Liu, Chao Wang, Zhao Huang, Liang Chu, Hui-fang Liang, Bi-xiang Zhang, Xiao-ping Chen

**Affiliations:** Hepatic Surgery Centre, Tongji Hospital, Tongji Medical College, Huazhong University of Science and Technology, 430030 Wuhan, Hubei China

## Abstract

18β-Glycyrrhetinic acid (GA) is the active ingredient of the traditional Chinese medicine, *Glycyrrhrzae Radix et Rhizoma*. Here, we explored the effects of GA on hepatocellular carcinoma (HCC) *in vitro* and *in vivo* and the underlying molecular mechanisms. We confirmed that GA suppressed proliferation of various HCC cell lines. Treatment of GA caused G0/G1 arrest, apoptosis and autophagy in HCC cells. GA-induced apoptosis and autophagy were mainly due to the unfolded protein response. We compared the roles of the ATF4/CHOP and IRE1α/XBP1s UPR pathways, which were both induced by GA. The ATF4/CHOP cascade induced autophagy and was indispensable for the induction of apoptosis in GA-treated HCC cells. In contrast, the IRE1α/XBP1s cascade protected HCC cells from apoptosis *in vitro* and *in vivo* induced by GA. Despite this, activation of autophagy protected HCC cells from apoptosis induced by GA. We concluded that pharmacological inhibition of autophagy or IRE1α may be of benefit to enhance the antitumor activity of GA.

## Introduction

Hepatocellular carcinoma (HCC) is the sixth most common malignant tumor, and the third leading cause of cancer mortality worldwide. Despite the introduction of new chemotherapeutic drugs, surgery remains the most effective way to treat HCC. However, surgery is limited by a high incidence of recurrence and intrahepatic/extrahepatic metastasis^1–3^.

Glycyrrhizin and glycyrrhetinic acid (GA) are the most important chemical components of the traditional Chinese medicine, *Glycyrrhrzae Radix et Rhizoma*^4^. They have long been used as hepatoprotective drugs to treat chronic hepatitis in China due to their anti-inflammatory and anti-viral effects^4^. GA is of benefit in the treatment of several kinds of cancer, including non-small-cell lung cancer, pituitary adenoma, breast cancer, epithelial ovarian carcinoma, and HCC^5–9^. The potential mechanisms of the anti-cancer activity of GA involve anti-proliferative/pro-apoptotic and/or anti-invasive/anti-metastatic activities^10^. GA produces its cytotoxic effect through several mechanisms, including inhibition of nuclear factor-κB, protein kinase C, Ras, and other anti-apoptotic proteins, or activation of the BH3 interacting-domain, kinase inhibitors, caspases, and other proapoptotic proteins^10^. However, the link between endoplasmic reticulum (ER) stress and apoptosis induced by GA in HCC needs to be fully elucidated.

The ER is the largest intracellular endomembrane system. It is responsible for protein synthesis, folding and maturation^11^. When cells are subjected to continuous chemical stimulation, oxidative stress, or calcium overload, intracellular proteins are damaged and the ER is challenged by accumulation of misfolded proteins. To prevent the ER from being overwhelmed, cells activate a series of adaptive mechanisms to compensate, which together are termed the unfolded protein response (UPR)^12^. The UPR is initiated by three signaling proteins named inositol-requiring protein (IRE)-1α, protein kinaseRNA-like ER kinase (PERK), and activating transcription factor(ATF)6; all of which are localized on ER membranes. Under ER stress, activation of the UPR reduces the unfolded protein load through several pro-survival mechanisms, including ER-associated degradation, reduction in the synthesis of new proteins, and the expansion of ER membranes. If ER stress cannot be quickly reversed, the UPR eliminates these cells by apoptosis^13^.

Autophagy is a major catabolic process that delivers proteins, cytoplasmic components, and organelles to lysosomes for degradation and recycling. It is essential for survival, differentiation, development, and homeostasis^14^. Autophagy principally serves to protect organisms against diverse pathologies, including cancer. In some contexts, autophagy suppresses tumorigenesis, although in most cases, autophagy facilitates tumorigenesis^14,15^. GA is able to induce both ER stress and autophagy in cancer cells; however, the contribution of these two processes remains a subject of interest^16–18^.

In the present study, we investigated the role of UPR proteins in mediating the effects of GA in HCC cell lines *in vitro* and *in vivo*. The ATF4/CHOP and IRE1α/XBP1s pathways had contrasting effects in GA-treated cells. GA-induced ATF4/CHOP activity was indispensable for both apoptosis and autophagy, whereas the IRE1α/XBP1s cascade limited apoptosis. Thus, induction of different UPR factors had contrasting actions on HCC cell biology.

## Results

### GA inhibits proliferation of HCC cell lines *in vitro* and *in vivo*

**Figure 1:**
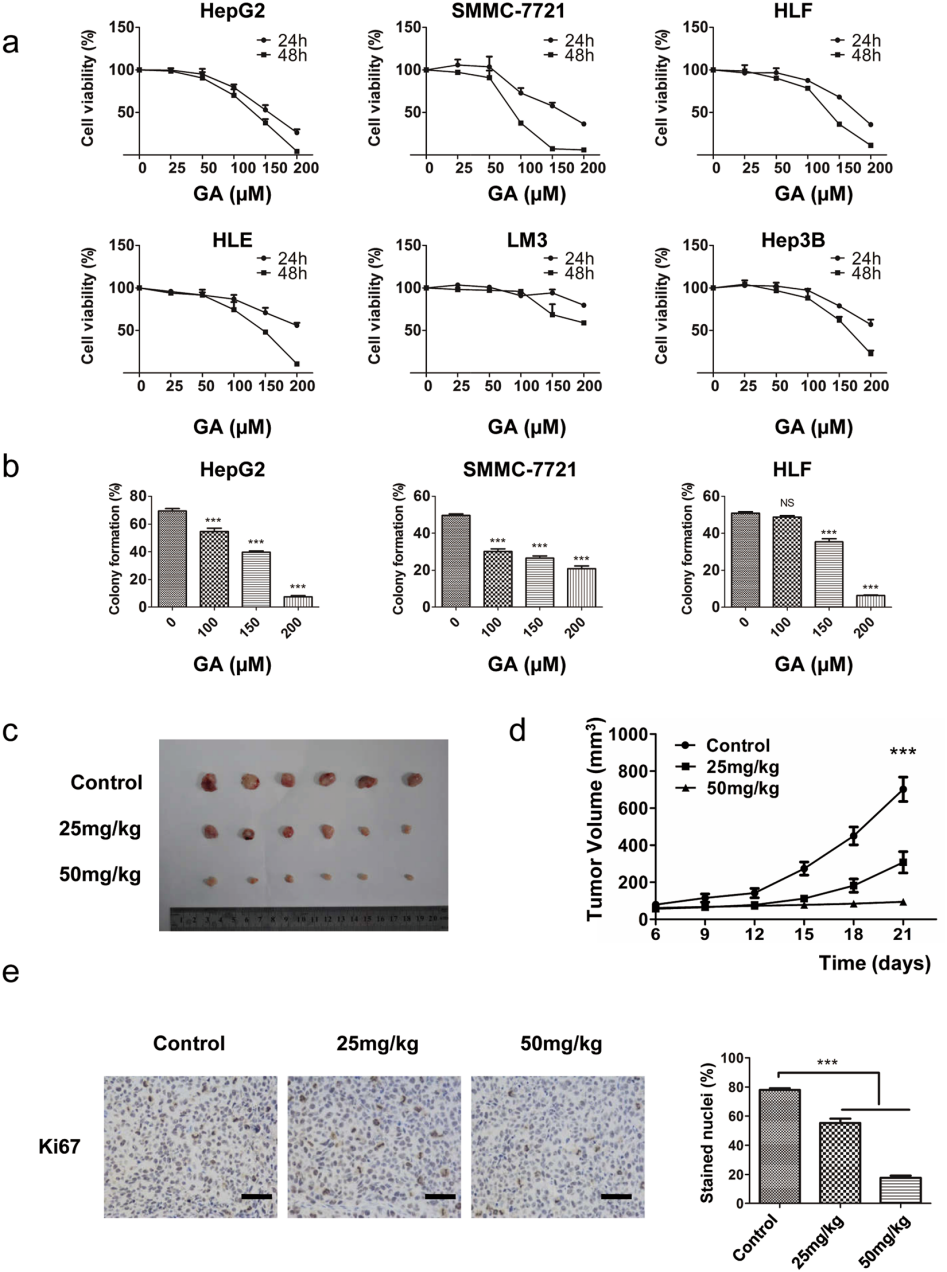
GA suppresses proliferation of HCC cells *in vitro* and *in vivo*. (**a**) HepG2, SMMC-7721, HLF, HLE, LM3 and Hep3B cells were treated with 0, 25, 50,100,150, or 200 μM GA for 24 and 48 h, and cell viability was calculated by the CCK-8 assay. (**b**) For the colony formation assay, HepG2, SMMC-7721, and HLF cells were treated with the indicated concentrations of GA for 36 h and then in complete medium for 12 days. HepG2 cells were subcutaneously injected into the mouse left axillary fossa. Drug administration was started 1 day after tumor implantation. Tumor volume was calculated every 3 days from day 6. (**c**) Representative images of subcutaneous tumors in GA-treated groups and the control group. (**d**) Tumor growth curve. (**e**) Immunohistochemical staining of Ki67 in control and GA-treated groups. Scale bar, 50 µm. Results are presented as the mean ± SEM from three independent experiments. *p < 0.05, **p < 0.01, ***p < 0.001.

We first assessed *in vitro* anti-proliferative activity of GA using the Cell Counting Kit-8 (CCK-8) assay on a panel of human HCC cell lines including HepG2, SMMC-7721, HLF, HLE, LM3 and Hep3B. Incubation of cells with increasing concentrations of GA for 24 or 48 h led to reduction in viability (Fig. 1a). The average inhibition by 150 µM GA treatment for 24 h was 46.2, 45.1, 32.1, 27.1, 5.8 and 21.3% in HepG2, SMMC-7721, HLF, HLE, LM3 and Hep3B cells, respectively. IC_50_ values for these HCC cells for 48 h were 124.0 ± 5.0, 88.3 ± 2.5, 131.5 ± 4.5, 137.3 ± 6.5, 218.0 ± 38.0, and 162.0 ± 3.5 µM, respectively (Supplementary Fig. 1a). GA treatment for 24 and 48 h had a slight effect on 7701 normal liver cells (Supplementary Fig. 1b). HepG2, HLF, and SMMC-7721 cells were more sensitive to GA than LM3, Hep3B, and HLE cells. These three HCC cell lines were selected for subsequent studies. To test the effect of GA on HCC cell tumorigenicity, colony formation assays were conducted. GA reduced the numbers of colonies of all HCC cell lines tested in a dose-dependent manner (Fig. 1b and Supplementary Fig. 1c). These data demonstrated that GA reduced HCC cell proliferation in a dose-dependent manner *in vitro*.

We explored the effect of GA on HCC cell lines *in vivo*. HepG2 cells were subcutaneously injected into nude mice and tumor growth was assessed after 21 days. Xenograft tumor growth was significantly inhibited by GA treatment at a dose of 25 or 50 mg/kg in comparison with the control group (Fig. 1c,d and Supplementary Fig. 1d). However, there was no significant body weight change between the control and GA treatment groups (Supplementary Fig. 1e). The reduction in tumor size in mice treated with GA was associated with a reduction in Ki67 expression and increased expression of cleaved caspase-3 (Fig. 1e and Supplementary Fig. 1f). Collectively, these data suggested that GA potently suppressed the growth of HCC cells *in vivo*.

### GA induces cell cycle G0/G1 arrest and apoptosis in HCC cells

**Figure 2:**
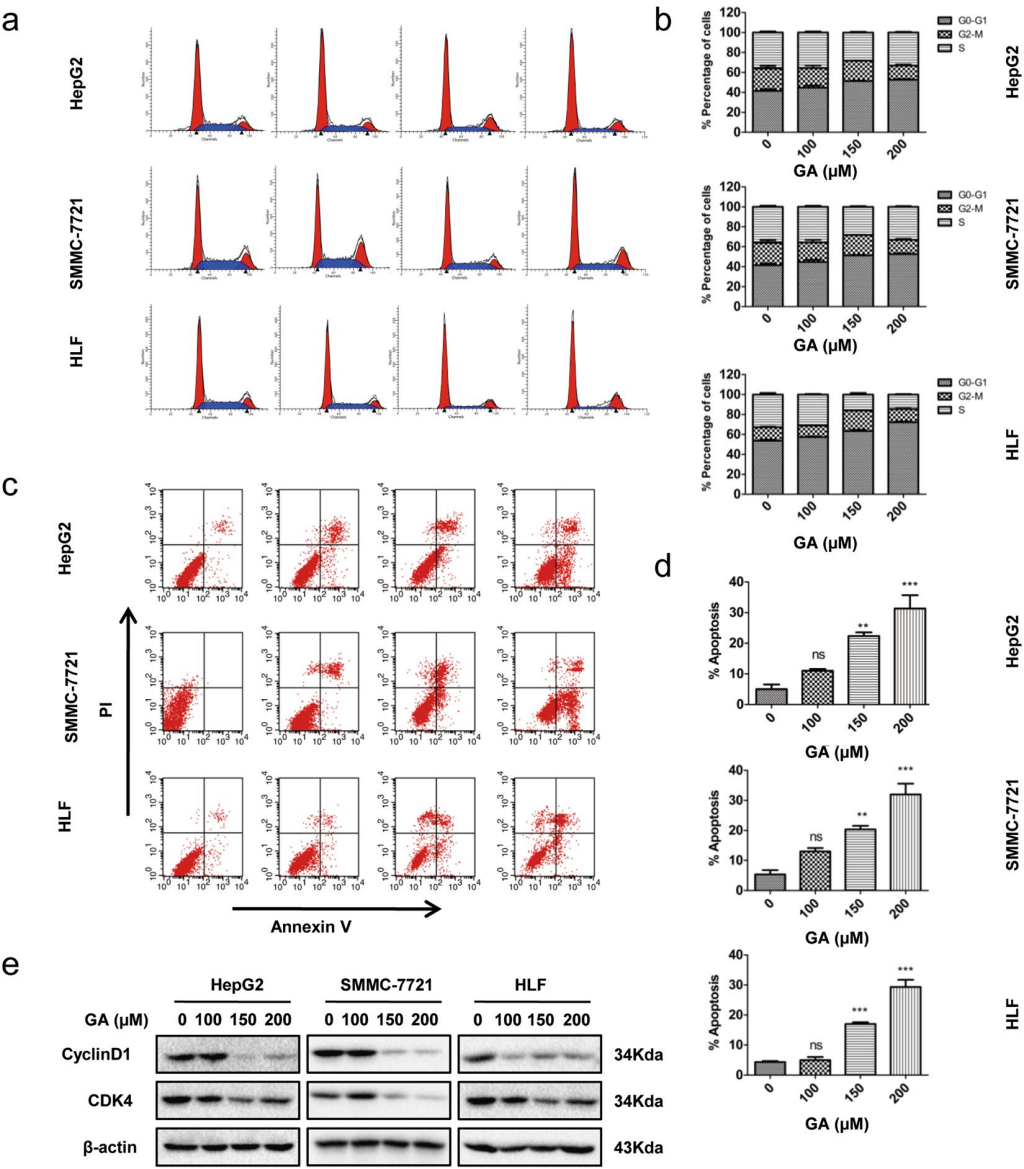
GA induces G0/G1 arrest and apoptosis in HCC cell lines. (**a**,**b**) For the cell cycle assay, HepG2, SMMC-7721, and HLF cells were treated with indicated concentrations of GA for 24 h, and the cell cycle distribution was examined by flow cytometry. (**c**,**d**) Cells were treated with indicated concentrations of GA for 48 h, and apoptosis was assessed by flow cytometry. (**e**) Western blot analysis of the expression of cell cycle-related proteins in cells followed treatment with GA for 24 h. The data are presented as the mean ± SEM from three independent experiments. *p < 0.05, **p < 0.01, ***p < 0.001.

To verify the possible mechanism of cell proliferation inhibition, we investigated the effects of GA on the cell cycle and apoptosis. Three HCC cell lines were treated with increasing concentrations (0, 100, 150, or 200 μM) of GA for 24 or 48 h, followed by measurement of the cell cycle and apoptosis by flow cytometry. We observed that GA induced G0/G1 arrest in three HCC cancer cell lines in a dose-dependent manner (Fig. 2a,b). This was associated with a significant increase in the number of apoptotic cells (Fig. 2c,d). After treatment with GA for 48 h, sub-G1 accumulation was increased significantly in HepG2 and HLF cells (Supplementary Fig. 2a). To confirm that GA promoted tumor cell death by apoptosis, we used a pan-caspase inhibitor, Z-VAD-FMK, to inhibit apoptosis. The CCK8 assay showed that GA-induced cell death was significantly reversed by Z-VAD-FMK (Supplementary Fig. 2c). This suggested that GA induced HCC cell death partially by apoptosis. Western blot analysis revealed that treatment with GA inhibited expression of cell cycle related proteins such as cyclin D1 and cyclin-dependent kinase (CDK)4 in these cells in a dose-dependent manner (Fig. 2e). All these findings indicated that GA triggered G0/G1 phase arrest and induced apoptosis in HCC cells.

### GA elicits autophagy flux in HCC cells

**Figure 3:**
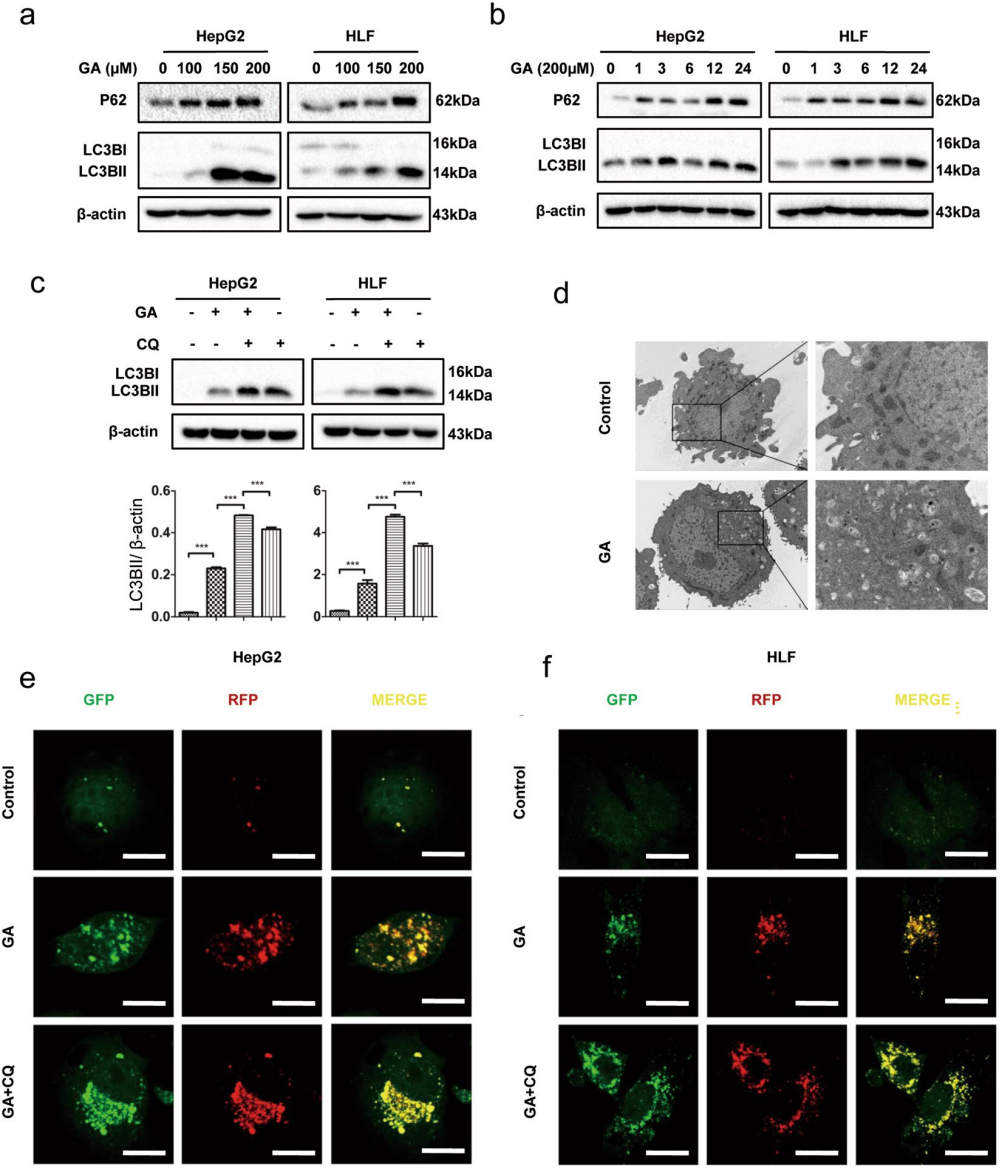
GA promotes autophagy flux in HCC cell lines. (**a**) HepG2 and HLF cells were treated with the indicated concentrations of GA for 24 h, and western blotting was performed for the autophagy marker LC3B and P62. (**b**) Cells were treated with 200 µM GA for the indicated periods, LC3B and P62 were analyzed by western blotting. (**c**) Cells were treated with GA for 24 h with or without pretreatment with CQ for 1 h. LC3B was analyzed by western blotting. Densitometry of LC3BII in each cell line. (**d**) HepG2 cells were treated with or without 200 μM GA for 24 h and analyzed by transmission electron microscopy. (**e**) HepG2 and HLF cells were transfected with mRFP–GFP-tagged LC3 for 48 h, and treated with GA for 24 h with or without pretreatment with CQ. Images were taken with a confocal microscope. Scale bar, 10 µm.

Autophagy has long been associated with apoptosis and the relationship is context-dependent^19,20^. To investigate further the effect of GA-induced autophagy on apoptosis, we studied whether GA elicited autophagy in HCC cells. Microtubule-associated protein light chain (LC)3 was used to monitor autophagy by detecting conversion from LC3BI to LC3BII, which is localized on the autophogosomal membranes^21^. HepG2 and HLF cells were treated with increasing concentrations of GA (0, 100, 150, or 200 µM) for 24 h. GA-treated cells showed by western blotting the detectable conversion of LC3BI to LC3BII (Fig. 3a). Upon GA treatment at 200 µM, LC3B conversion was steadily increased in a time-dependent manner (Fig. 3b). Unexpectedly, we observed that another autophagy marker, P62, was also up-regulated in a dose- and time-dependent manner instead of being degraded by autophagy (Fig. 3a,b). HepG2 cells were cultured with or without GA for 24 h and subjected to transmission electron microscopy. We observed a significantly larger number of autophagosomes in the cytosol of HepG2 cells treated with GA compared to the control group (Fig. 3d).

To confirm that this phenomenon was associated with an increase in the autophagy flux in HCC cells, we compared the amount of LC3B in the absence or presence of lysosomal inhibitors. Chloroquine (CQ) was used to block autophagy by impairing lysosomal function. HepG2 and HLF cells were pretreated with 10 µM CQ for 30 min and then treated with GA for a further 24 h. Accumulation of LC3BII was significantly increased in the presence of CQ (Fig. 3c). To assess autophagy flux, HepG2 and HLF cells were infected with lentivirus coding for mRFP–GF–LC3 fusion protein. In the acidic environment of the lysosomes, GFP is quenched but RFP is preserved. Thus, autophagosomes can be identified as yellow (mRFP and GFP) and autolysosomes identified as red (mRFP only)^22^. The mRFP–GFP–LC3-expressing HepG2 and HLF cell lines were incubated in the presence or absence of GA or CQ. After 24 h, we observed a significant increase in the appearance of yellow (autophagosomes) and red (autolysosomal) puncta in GA-treated cells compared to control cells. In the presence of CQ, the number of GA-induced puncta was greater than with GA treatment alone and almost all were yellow, in keeping with a loss of lysosmal function (Fig. 3e,f and Supplementary Fig. 5). GA also promoted LC3B accumulation *in vivo* (Supplementary Fig. 2b). Taken together, these data demonstrated that GA induced autophagy *in vitro* and *in vivo* in HCC cells.

### Autophagy inhibition augments apoptotic cell death induced by GA

**Figure 4:**
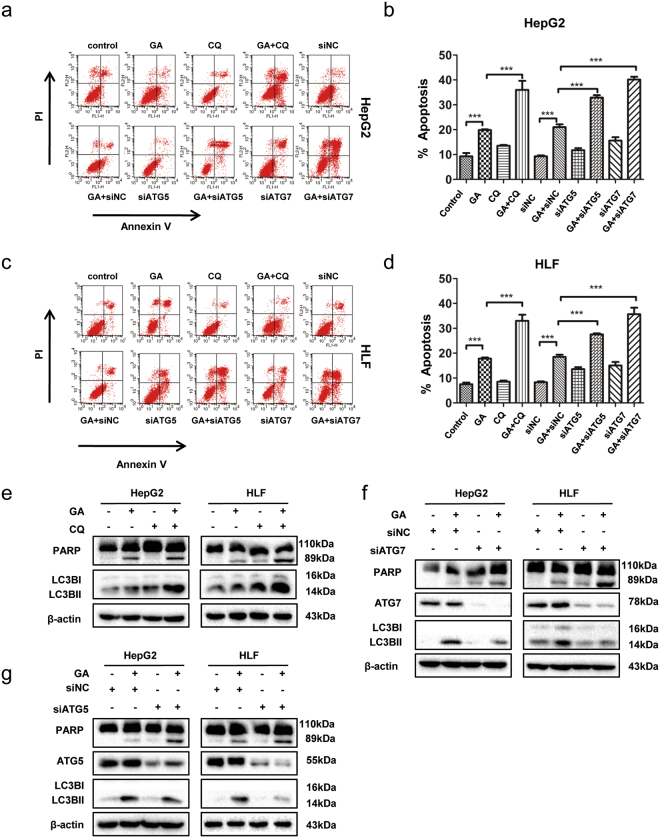
Autophagy inhibition augments apoptotic cell death induced by GA. (**a**–**d**) HepG2 and HLF cells were transfected with ATG5/7 siRNA and negative control for 48 h, or pretreated with or without CQ for 1 h and then with GA (200 µM) for 24 h. Apoptosis was examined by FACS. (**e**) Cells were pretreated with or without CQ for 1 h prior to treatment with GA for 24 h. PARP was analyzed by western blotting. (**f**,**g**) Cells were transfected with ATG5/7 siRNA and the negative control for 48 h prior to GA treatment for 24 h. PARP was analyzed by western blotting. Data presented as mean ± SEM from three independent experiments. *p < 0.05, **p < 0.01, ***p < 0.001.

We determined whether GA-induced autophagy was cytoprotective or cytotoxic. Autophagy was inhibited pharmacologically using CQ or genetically by siRNA against autophagy protein ATG5 and ATG7. As expected, CQ increased accumulation of LC3BII (Fig. 4e). Treatment with CQ significantly increased GA-induced apoptosis in HepG2 and HLF cells (Fig. 4a–d). This was associated with increased cleaved poly (ADP-ribose) polymerase (PARP) in GA/CQ-treated cells compared with GA-treated cells when measured by western blotting (Fig. 4e). To confirm these results, we used siRNA to silence ATG5 and ATG7 expression, which blocked autophagy initiation. Western blotting showed that that ATG7 and ATG5 were efficiently decreased (Supplementary Fig. 2d). The expression of the autophagy marker LC3BII was reduced and cleaved PARP accumulated in ATG-silenced HepG2 and HLF cells with GA treatment (Fig. 4f,g). These changes were associated with a significant increase in apoptosis (Fig. 4a–d). To investigate further whether autophagy mediated P62 degradation, we used the autophagy inhibitor, CQ or ATG7 siRNA, to block autophagy. P62 expression was increased further by autophagy inhibition (Supplementary Fig. 3a). This suggested that autophagy was responsible for degradation of some P62. To prove that autophagy was antagonistic for apoptosis, we used a classic autophagy inducer, rapamycin, which promoted autophagy by inhibiting the activity of the mammalian target of the rapamycin complex 1^23^. HepG2 cells were treated with GA in the presence or absence of rapamycin for 24 h and then subjected to the CCK8 assay and western blotting. We observed that rapamycin partially reduced GA-induced cell death and expression of cleaved PARP (Supplementary Fig. 3b,c). Collectively, these data indicated that the GA-induced autophagic response played a cytoprotective role in counteracting apoptosis.

### GA induces ER stress via ROS production and promotes apoptosis via CHOP induction

**Figure 5:**
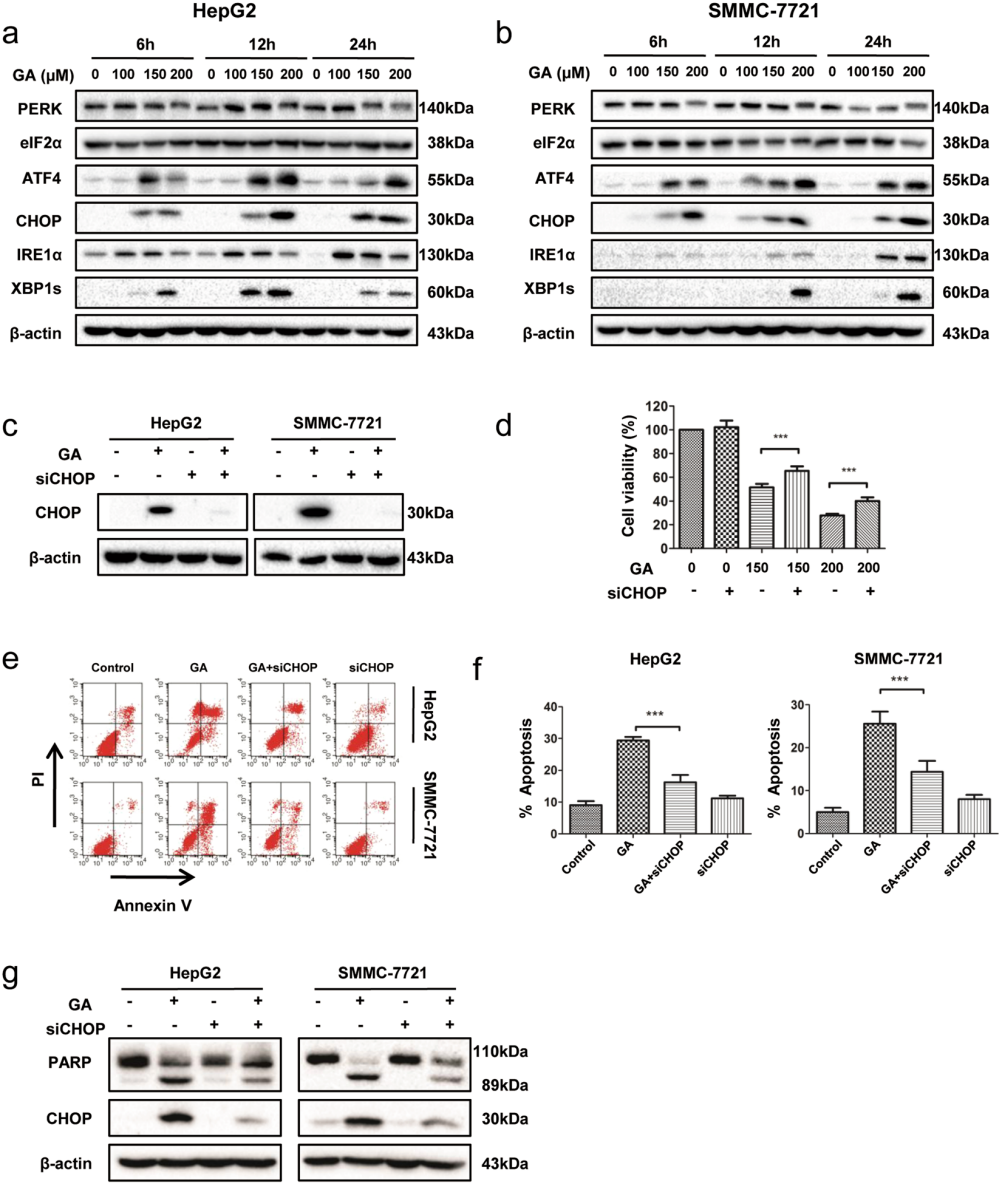
GA induces CHOP-dependent apoptosis in HCC cell lines. (**a**,**b**) HepG2 and SMMC-7721 cells were treated with GA for 6, 12, and 24 h. ER stress-related proteins were detected by western blotting. (**c**) HepG2 and SMMC-7721 cells were transfected with CHOP siRNA and the negative control for 48 h, and then with GA (200 µM) for 24 h. Western blotting of CHOP expression was examined. (**d**) HepG2 cells were transfected with CHOP siRNA and the negative control for 48 h prior to GA treatment for 24 h. Cell viability was measured by the CCK-8 assay. (**e**–**g**) HepG2 and SMMC-7721 cells were transfected with siRNA for 48 h and then with GA (200 µM) for 36 h. Apoptosis was measured by FACS with annexin V/propidium iodide staining (**e**,**f**). Expression of the apoptosis marker PARP was examined by western blotting (**g**).

ER stress, a common cellular stress response, is triggered by a variety of conditions that disturb cellular homeostasis and induces cell apoptosis and autophagy^24^. UPR-related proteins were detected in GA-treated cells by western blotting. Upon GA treatment with increasing doses (0, 100, 150, or 200 μM), UPR-related proteins including ATF4, CCAAT-enhancer-binding protein homologous protein (CHOP), IRE-1α, and X-box binding protein (XBP)-1s (XBP1s) were induced in SMMC-7721 and HepG2 cell lines in a dose- and time-dependent manner (Fig. 5a,b and Supplementary Fig. 4a,b). GA increased the expression of CHOP *in vivo* (Supplementary Fig. 2b). Previous studies have reported that reactive oxygen species (ROS) generation could trigger ER stress^25,26^. Pretreatment with ROS inhibitor N-acetylcysteinepartially blocked expression of ATF4, CHOP, and IRE1α in GA-treated HepG2 cells (Supplementary Fig. 2e). These data indicated that GA induced ER stress via ROS production.

We investigated whether GA-induced apoptosis resulted from ER stress. The role of CHOP has been established in ER stress-induced apoptosis^27^. To verify the role of CHOP in GA-induced apoptosis, we blocked GA-induced CHOP expression using siRNA in HepG2 and SMMC-7721 cell lines (Fig. 5c). We measured cell viability (Fig. 5d) and apoptosis (Fig. 5e,f) and found that induction of apoptosis by GA was significantly impaired in cell lines treated with CHOP siRNA. Western blotting showed that after CHOP silencing, cleaved PARP was significantly reduced in response to GA treatment (Fig. 5g). We concluded that induction of CHOP was necessary for apoptosis induced by GA in HCC cells.

### GA induces autophagy via the ATF4/CHOP cascade

**Figure 6:**
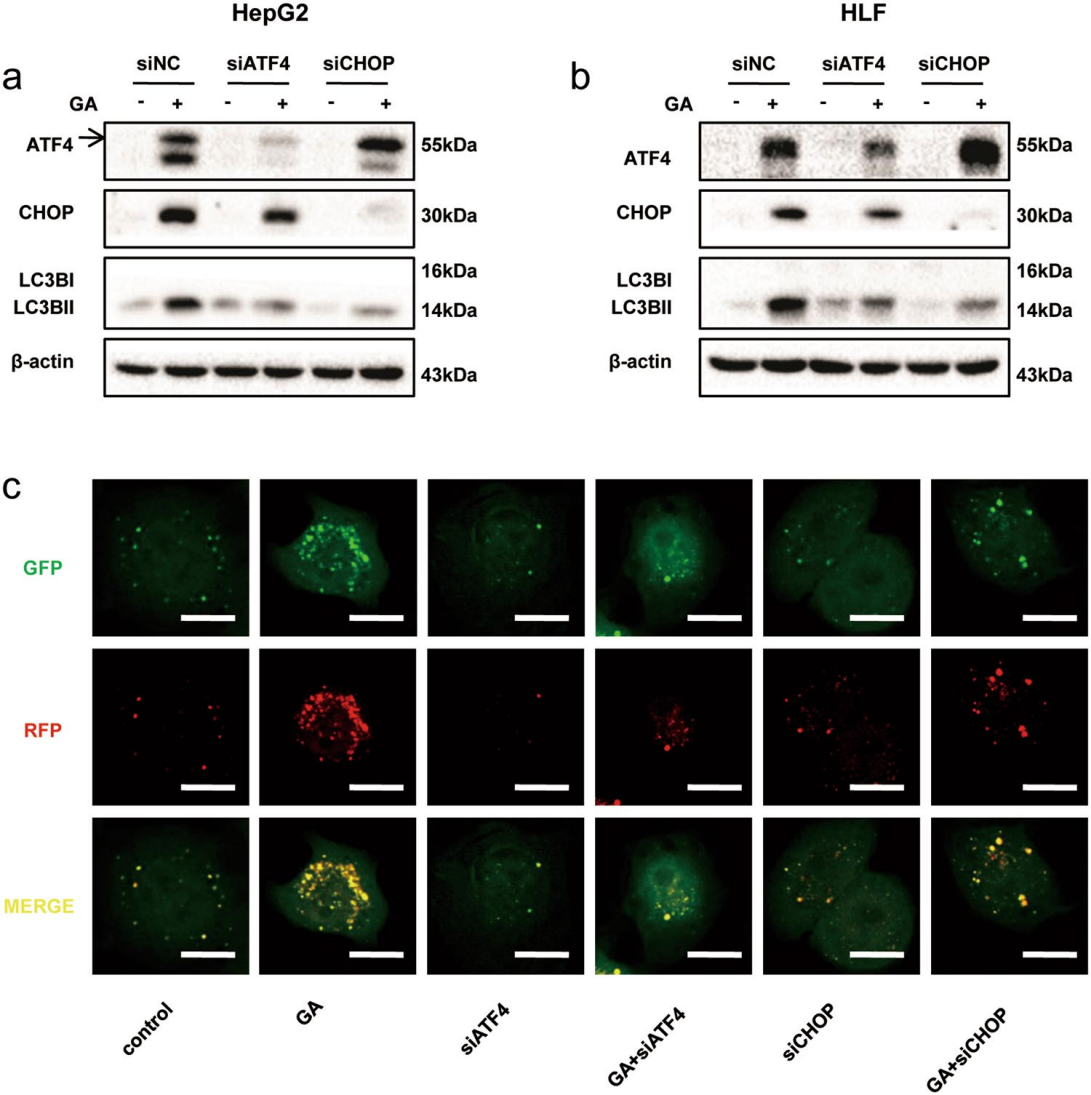
GA induces autophagy through the ATF4/CHOP cascade. (**a**,**b**) HepG2 and HLF cells were transfected with ATF4/CHOP siRNA and the negative control for 48 h, and then treated with GA for 24 h. ATF4, CHOP, and LC3B were analyzed by western blotting. (**c**) mRFP-GFP-tagged LC3 stably transfected HepG2 cells were transfected with ATF4/CHOP siRNA and the negative control for 48 h, and then treated with GA for 24 h. Images were taken with a confocal microscope. Scale bar, 10 µm.

The link between ER stress and autophagy has been substantiated by numerous excellent studies^28,29^. To investigate whether GA-induced autophagy resulted from ER stress, we used siRNA to silence expression ATF4 and its downstream target, CHOP. HepG2 and HLF cells were transfected with siRNA for 48 h and treated with GA for a further 24 h. We found a reduction in GA-induced CHOP expression in cells treated with ATF4 siRNA but an increase in GA-induced ATF4 expression in cells treated with CHOP siRNA. In both cases, the level of LC3BII was significantly decreased in the absence of either ATF4 or CHOP (Fig. 6a,b). Consistent with these data, in GA-treated ATF4- and CHOP-deficient cells, GFP–LC3 puncta formation was markedly decreased compared to that in GA-treated control cells (Fig. 6c). We concluded that GA induced autophagy via the ATF4/CHOP cascade in HCC cells.

To explore further the effect of autophagy on ER stress, we performed western blotting on GA-treated HLF cells. Inhibition of autophagy using CQ or ATG5/7 siRNA resulted in induction of the ER-stress-related protein, IRE-1α. In contrast, CQ or ATG5/7 siRNA treatment had little effect on CHOP expression (Supplementary Fig. 3d).

### IRE1α protects HCC cells from apoptosis by GA *in vitro* and *in vivo*

**Figure 7:**
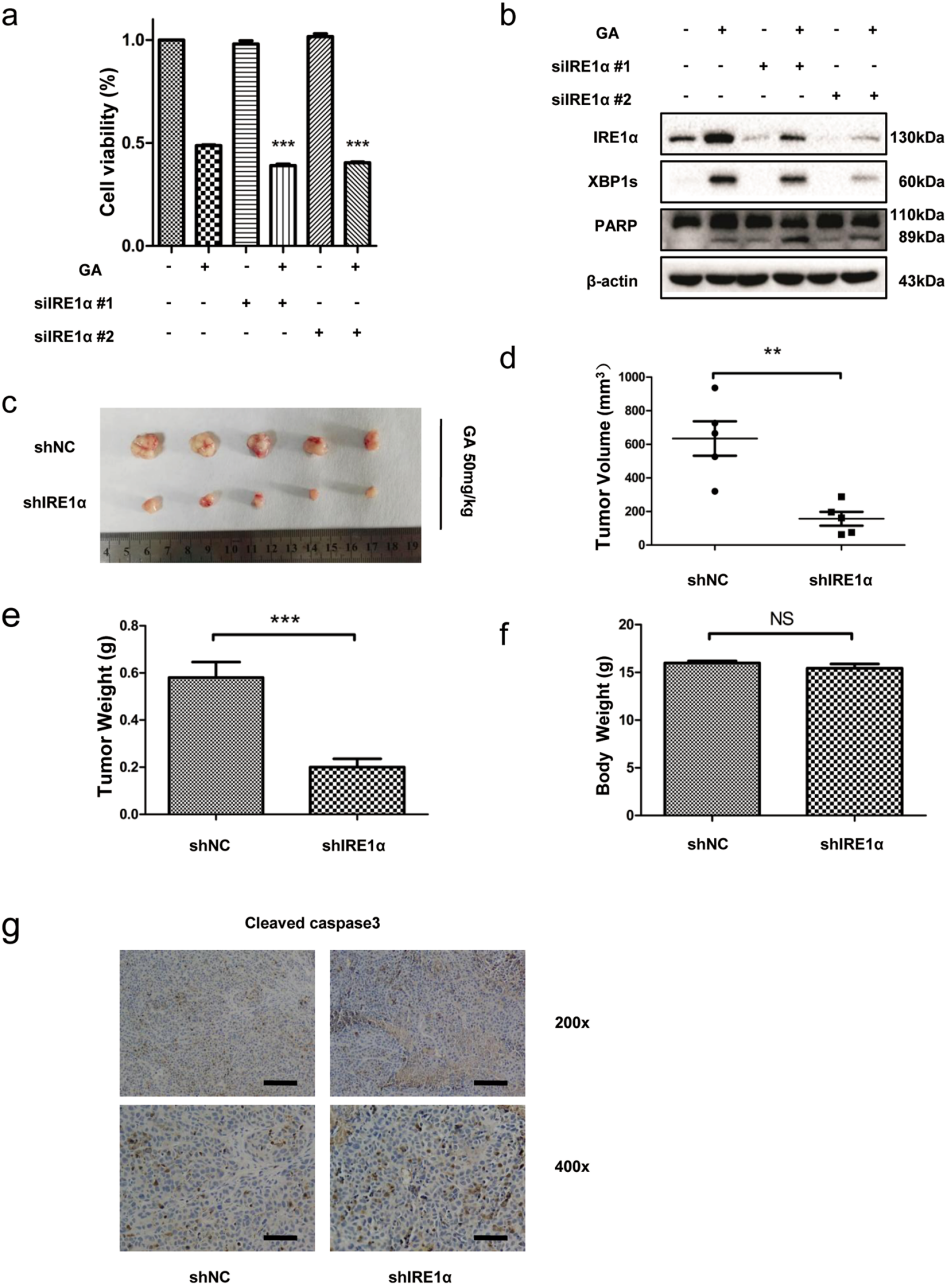
IRE-1α protects HCC cells from apoptosis by GA *in vitro* and *in vivo*. HepG2 cells were transfected with IRE-1α siRNA or the negative control for 48 h, and then treated with GA for 24 h. Cell viability was calculated by the CCK-8 assay (**a**) and western blotting was performed for IRE-1α, XBP1s, and PARP (**b**). HepG2-shNC and HepG2-shIRE1α cells were subcutaneously injected into the left axillary fossa of mice. Drug administration was started 1 day after tumor implantation. (**c**) Representative images of subcutaneous tumors in HepG2-shNC and HepG2-shIRE1α mice. (**d**) Tumor volume. (**e**) Tumor weight. (**f**) Body weight of mice. (**g**) Immunohistochemical staining of cleavedcaspase-3 in different groups. Scale bar, 100 µm (top). Scale bar, 50 µm (below).

We investigated the role of another UPR factor, IRE1α. We used siRNA to silence IRE-1α expression induced by GA. Expression of IRE-1α and XBP-1s was significantly decreased after transfection with siRNA (Fig. 7b). In contrast with inhibition of CHOP, IRE-1α-silenced HepG2 cells had reduced cell viability (Fig. 7a) and increased cleaved PARP after incubation with GA compared with control cells (Fig. 7b). To confirm these results, we performed an *in vivo* study. HepG2-shNC and HepG2-shIRE1α cells (2 × 10^6^) were injected subcutaneously into the left axillary fossa of mice (n = 5). Five weeks later the mice were sacrificed and tumor volume and weight were calculated. Knockdown of IRE-1α resulted in a significant reduction in tumor volume and weight by GA treatment (Fig. 7c–e), but had no effect on body weight (Fig. 7f). Immunohistochemistry showed that expression of cleaved caspase-3 in the HepG2-shIRE1 group was increased (Fig. 7g). These findings suggested that IRE-1α was antagonistic to CHOP and limited the action of GA *in vitro* and *in vivo*.

## Discussion

GA is the active ingredient of the traditional Chinese medicine, *Glycyrrhrzae Radix et Rhizoma*, which has been used as hepatoprotective drugs in China and Japan^4^. Numerous studies have confirmed that GA has antitumor effects in breast cancer, non-small-cell lung cancer, and pituitary adenoma by triggering apoptosis, inhibiting proliferation, or suppressing metastasis^7,18,30^. In the present study, we showed that GA reduced cell viability, induced G0/G1 phase arrest, and promoted apoptosis and autophagy in HCC cell lines. However, GA-induced autophagy played a cytoprotective role in HCC cells. We found that GA activated several UPR pathways *in vitro* and *in vivo* that had contrasting effects on apoptosis. We found that GA-induced ATF4/CHOP activity was necessary for apoptosis and autophagy, whereas IRE-1α/XBP-1s limited apoptosis.

Autophagy is a conservative process that degrades intracellular proteins or organelles to maintain homeostasis^15^. As a promising, novel strategy for enhancing anti-tumor efficacy of chemotherapy drugs, it has been under extensive investigation^31^. A previous study reported that GA induced autophagy by activation of the ERK pathway^17^. In our study, we found that GA elicited autophagy in HCC cell lines by increasing the accumulation of LC3BII. Autophagy is generally linked with maintaining cell survival in the absence of nutrients, but in some studies it has been linked with cell death^20^. We found that autophagy played a role to limit the cytotoxic effects of GA. In non-small-cell lung cancer, autophagy induction by GA is initiated through the IRE-1α/JNK pathway^16^. In the present study, we observed that ATF4 and CHOP were both essential for GA-induced autophagy in HCC cells. As an upstream regulator of CHOP, ATF4 silencing significantly reduced expression of CHOP. This suggested that increasing CHOP expression mainly resulted from ATF4 induction, although it was not the sole factor. In addition, GA up-regulated the expression of ATF4 when CHOP expression was silenced. This suggested a possible feedback from CHOP to ATF4, and further suggested that GA-induced ATF4 expression promoted autophagy via its downstream CHOP instead of directly regulating autophagy. This was consistent with reports showing that CHOP directly increases expression of LC3B and ATG5 to promote autophagy at the transcriptional level^32^. GA induced CHOP expression in HepG2 and HLF cell lines at 6 and 3 h, respectively. The cells, however, did not undergo apoptosis until 24 h. Hence, CHOP may not always induce apoptotic cell death in HCC cell lines. After short-term exposure to GA, CHOP promoted survival by inducing autophagy. If GA treatment was prolonged, the cells inevitably underwent apoptosis by CHOP induction.

Previous studies have shown that GA induces apoptosis via the ROS/MAPK pathway in pituitary adenoma cells^7^ and through modulation of the AKT/FOXO3A/BIM pathway in human breast cancer^33^. In the current study, we focused on ER stress. We found that GA induced the UPR in HCC cells lines, including activation of the ATF4/CHOP and IRE-1α/XBP1s pathways. CHOP has been reported to mediate ER stress-related apoptosis by regulating mitochondrial apoptotic or extrinsic apoptotic pathways^34,35^. In our study, we found that GA induced apoptosis in HCC cells by CHOP induction. However, knockdown of CHOP expression may not totally block GA-induced apoptosis. This suggested that other mechanisms were also involved in GA-induced apoptosis. In contrast, the role played by IRE-1α is controversial. Phosphorylated IRE-1α is reported to induce apoptosis through recruitment of tumor necrosis factor receptor-associated factor-2 to its cytosolic domain, leading to the activation of c-JUN NH_2_*-*terminalkinases(JNKs)^27^. JNKs belong to the mitogen-activated protein kinases that are involved in regulation of cell proliferation, differentiation, and apoptosis. JNKs promote apoptosis through the coordinated regulation of extrinsic and intrinsic apoptotic pathways^36,37^. Conversely, IRE-1α may play a role in cell survival^38,39^. We found that in HCC cells, IRE-1α signaling contributed to cell survival *in vitro* and *in vivo*. Hence, we concluded that GA promoted apoptotic cell death in HCC cell lines by CHOP induction, while IRE-1α played an opposite role in GA-induced apoptosis.

To investigate further the effect of autophagy inhibition on ER stress, we used an autophagy inhibitor and siRNA to silence ATG5 and ATG7. Upon inhibition of autophagy, ER stress-related protein, IRE1α, was increased, while CHOP expression was unchanged. This suggested that autophagy partially relieved ER stress by eliminating damaged ER. However, ER-phagy may also be a selective process dependent on ER-resident receptors^40^.

In conclusion, our data demonstrated that GA suppressed the growth of HCC *in vitro* and *in vivo*. GA-induced ER stress in HCC cells activated the ATF4/CHOP and IRE-1α/XBP1s pathways to relieve the ER burden. The ATF4/CHOP signaling pathway mediated GA-induced cytoprotective autophagy and apoptosis, while IRE-1α contributed to survival in HCC cells. We concluded that CHOP played a dual role in determining cell fate in the crosstalk between autophagy and apoptosis. Collectively, GA has an anti-tumor effect, and inhibition of autophagy or IRE1α may enhance the anti-tumor efficacy.

## Materials and Methods

### Cell culture and reagents

Six hepatoma cell lines (HepG2, SMMC-7721, HLF, HLE, Hep3B, and LM3) were purchased from China Centre for Type Culture Collection (Wuhan, China). Cells were cultured in high glucose medium supplemented with 10% fetal bovine serum (Gibco) in a humidified atmosphere containing 5% CO_2_ at 37 °C. GA was purchased from Sigma-Aldrich (St. Louis, MO, USA) and dissolved in DMSO (Sigma-Aldrich).

### Cell viability assays

Cell viability was determined using the CCK-8 assay (Beyotime Institute of Biotechnology). Indicated cells (counted by a Cellometer Mini; Nexcelom Bioscience, Lawrence, MA, USA) were seeded in 96-well plates and treated with different concentrations of GA for 24 or 48 h to generate a dose-dependent curves. CCK-8 was added to the plates for 1–2 h to determine the OD_450_. IC_50_ was then calculated using GraphPad Prism, version 5.0.

### Colony formation assays

Indicated cells were plated in six-well plates and treated with medium containing various concentrations of GA for 36 h. Medium was replaced with fresh culture medium every 2 days. After 14 days, the plates were stained with 1% Crystal violet (Sigma-Aldrich) and photographed. Colonies were counted and analyzed using the Alpha Innotech Imaging system (Alphatron Asia, Singapore).

### Cell cycle and apoptosis assays

Flow cytometry analysis was performed as described previously^41^. For the cell cycle assay, indicated cells were treated with different concentrations of GA for 24 h and harvested for the following steps. A total of 1 × 10^6^ cells per sample were analyzed for cell cycle distribution on a FACS Aria Cell Cytometer (BD Biosciences, San Jose, CA, USA). For the apoptosis assay, 2 × 10^5^ cells per sample were harvested and tested. All data were analyzed using CellQuest software (BD Biosciences).

### RNA interference

RNA interference was used to knock down CHOP, ATG5, and ATG7. The siRNA oligonucleotides were used as follows:

CHOP: 5′ GGCTCAAGCAGGAAATCGA 3

ATG7: 5′ GGAGTCACAGCTCTTCCTT 3′

ATF4: 5′ GCCTAGGTCTCTTAGATGA3′

ATG5: 5′ CCTGAACAGAATCATCCTTAA 3′

IRE1α #1: 5′ CCAACAATCTACCCAAACA3′

IRE1α #1: 5′ GCACGTGAATTGATAGAGA 3′.

A scrambled siRNA was used as a negative control. The silencing efficiency was detected by western blotting. At 48 h after transfection, cells were treated with 200 µM GA.

### Transmission electron microscopy

Standard transmission electron microscopy was performed as previously described^42^. The cells were fixed and embedded. Thin sections (90 nm) were examined at 80 kV with a JEOL 1200EX transmission electron microscope. Approximately 15 cells were counted, and autophagosomes were defined as doublemembrane vacuoles.

### mRFP–GFP–LC3 puncta assay

Autophagy was examined by analyzing the formation of fluorescent puncta of autophagosomes in cells transfected with mRFP-GFP-LC3. Cells were cultured in 24-well plates and transfected with mRFP-GFP-LC3 lentivirus. At 48 h post-transfection, the cells were treated with 200 µM GA in the absence or presence of CQ. Fluorescence images of indicated cells were directly taken using an inverted confocal microscope (Olympus).

### Western blotting

Immunoblotting was performed as described previously^43^. Primary antibodies and their sources were as follows: β-actin and CDK4 (Santa Cruz Biotechnology, Santa Cruz, CA, USA); cyclinD1, ATG5, and ATG7 (Abcam, Cambridge, UK); cyclinB1, cleaved caspase-3, PARP, eIF2α, IRE1α, XBP1s, and LC3 (Cell Signaling Technology, Beverly, MA, USA); and ATF4 and CHOP (Proteintech Group, Chicago, IL, USA).

### Xenograft tumorigenicity assay

Animal studies were performed as described previously^44^. All *in vivo* studies were performed in compliance with the National Institutes of Health guidelines (NIH publication 86–23, revised 1985), and approved by the Committee on the Ethics of Animal Experiments of Tongji Medical College, Huazhong University of Science and Technology. HepG2 cells were subcutaneously injected into the left axillary fossa of mice. Drug administration started from day 1. GA (25 or 50 mg/kg) was injected intraperitoneally every day until the end of the study. Subcutaneous tumors were removed. Tumor volume was calculated using the following formula: tumor size (mm^3^) = (d2 × D)/2, where d and D are the shortest and longest diameters of the tumor, respectively.

### Statistical analysis

Data were analyzed using GraphPad Prism version 5.0. All experiments were performed at least three independent times, and the results were presented as the mean ± SEM. Comparisons between the different groups were evaluated using one-way analysis of variance, and *p* < 0.05 was considered statistically significant.

## Electronic supplementary material

Supplementary Information
