# Supplementary material for: 18β-Glycyrrhetinic-acid-mediated unfolded protein response induces autophagy and apoptosis in hepatocellular carcinoma
Source: Sci Rep. 2018 Jun 19;8:9365. doi: 10.1038/s41598-018-27142-5 (PMC6008326; doi:10.1038/s41598-018-27142-5)
Supplement: Supplementary file 1 — Supplementary Information [file 41598_2018_27142_MOESM1_ESM.docx]

**18β-glycyrrhetinic acid-mediated unfolded protein response induces autophagy and apoptosis in hepatocellular carcinoma**

Jin Chen^1^, Zhao-qi Zhang^1^, Jia Song^1^, Qiu-meng Liu^1^, Chao Wang^1^, Zhao Huang^1^, Liang Chu^1^, Hui-fang Liang^1^, Bi-xiang Zhang^1^, Xiao-ping Chen^1^

**Supplementary Fig. 1**

**
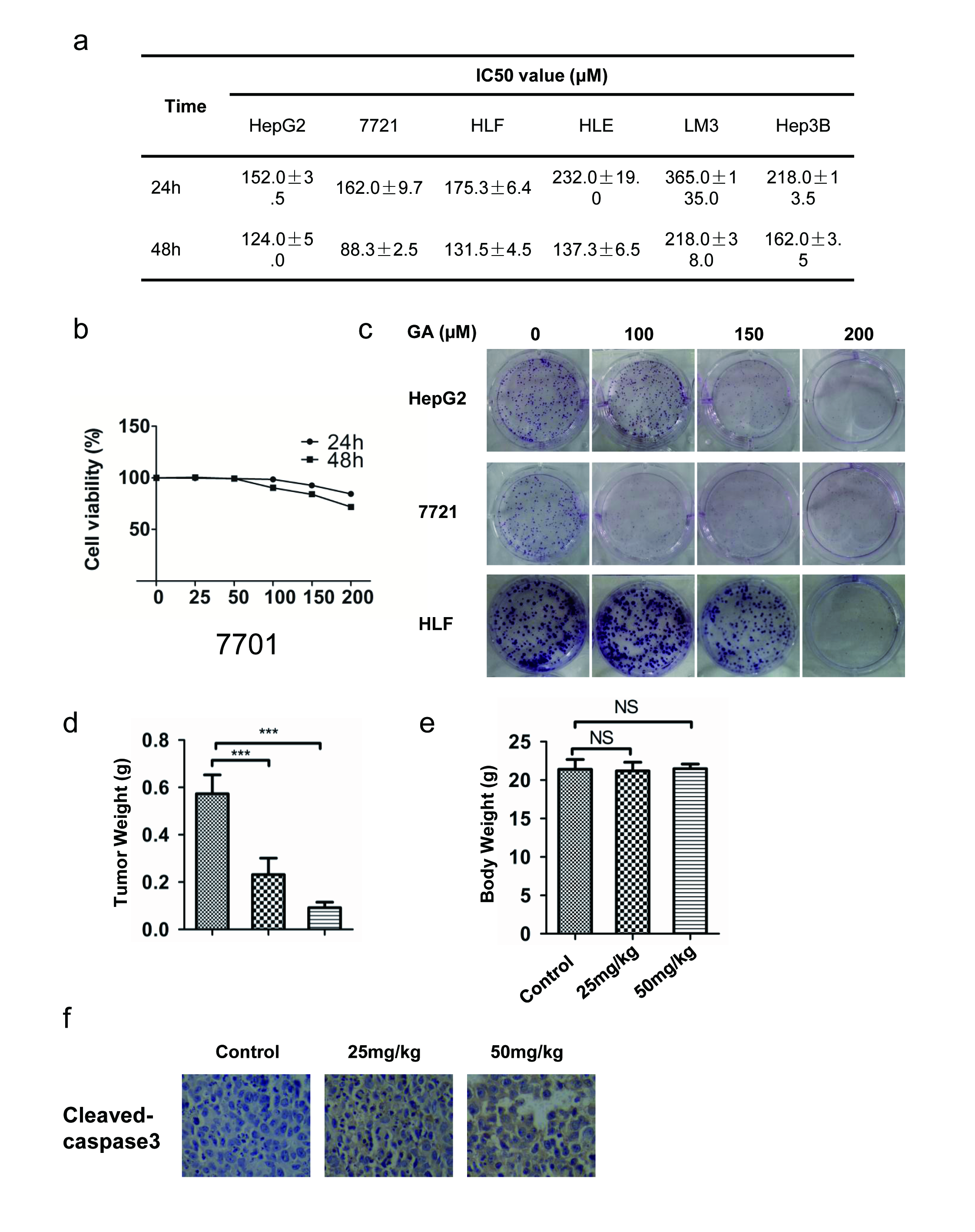
**

**Supplementary Fig. 2**

**
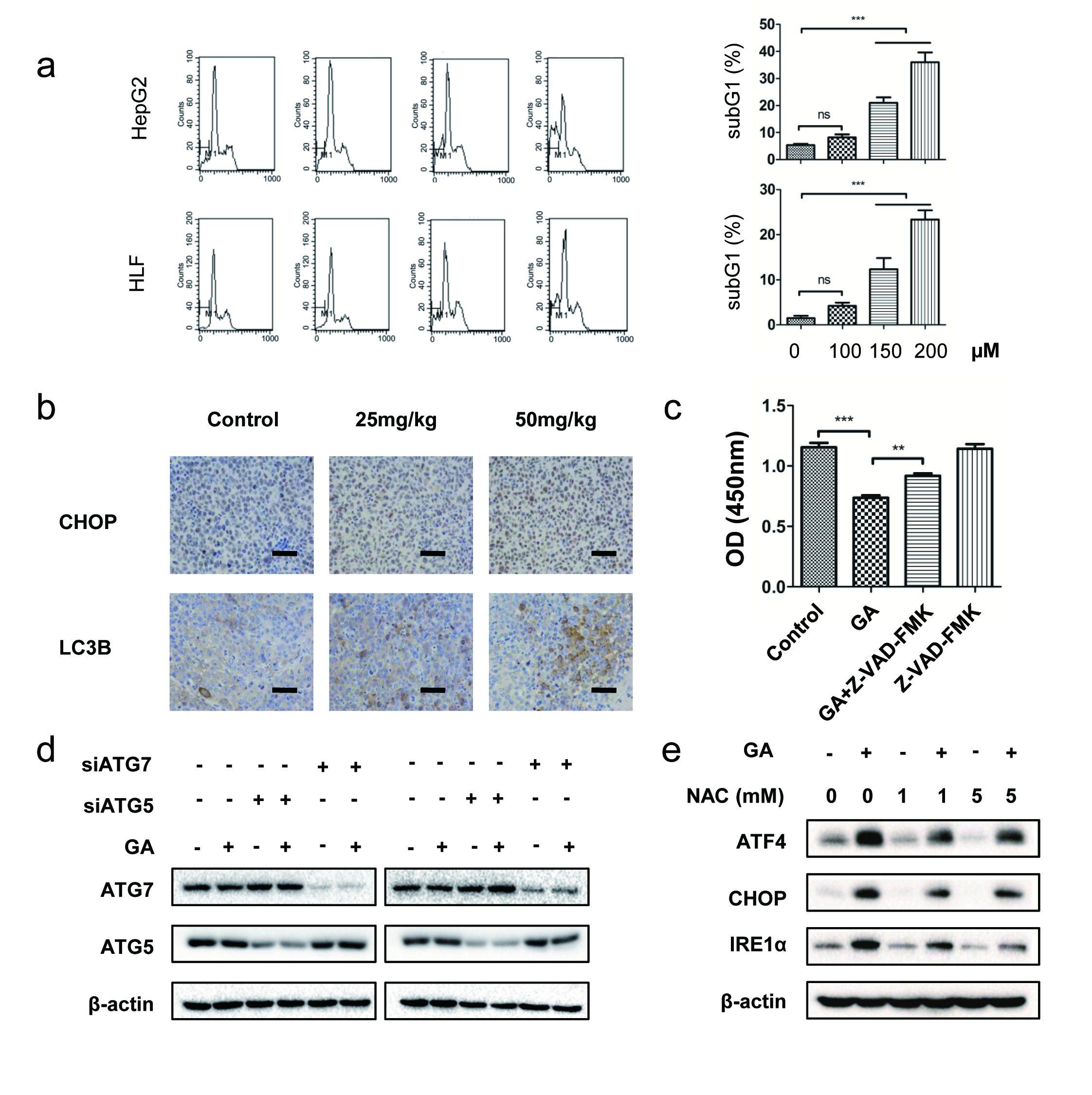
**

**Supplementary Fig. 3**

**
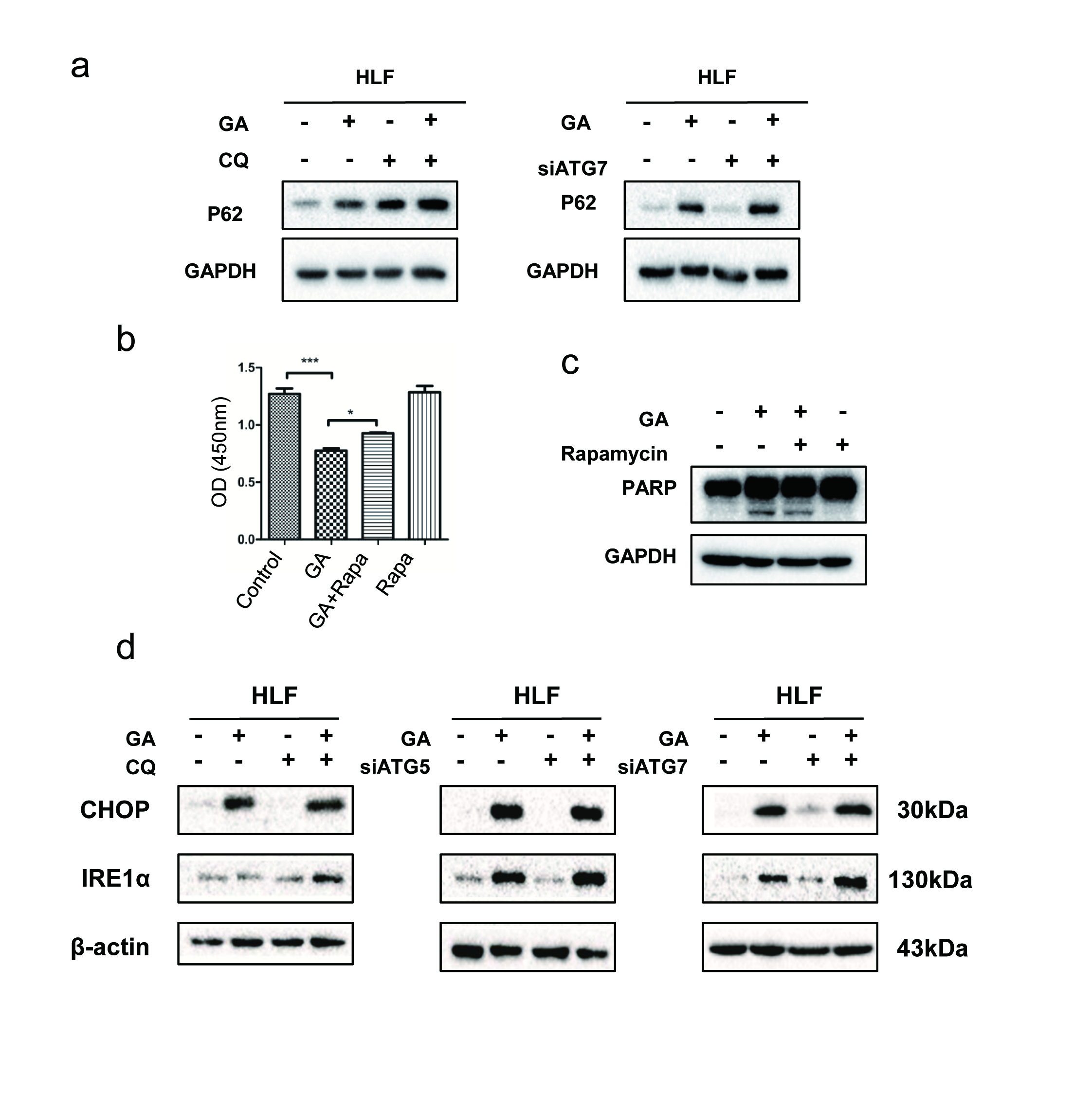
**

**Supplementary Fig. 4**

**
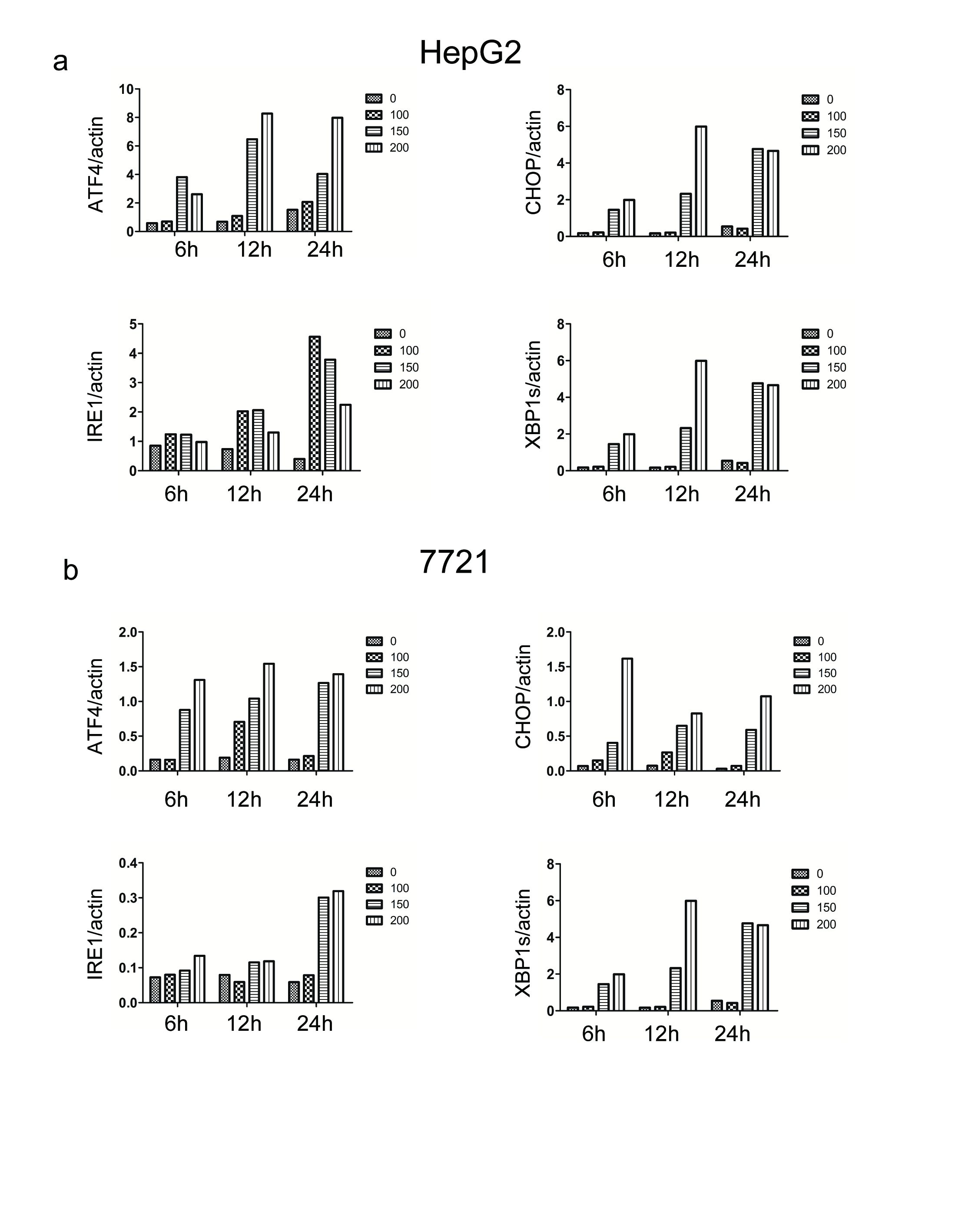
**

**Supplementary Fig. 5**

**
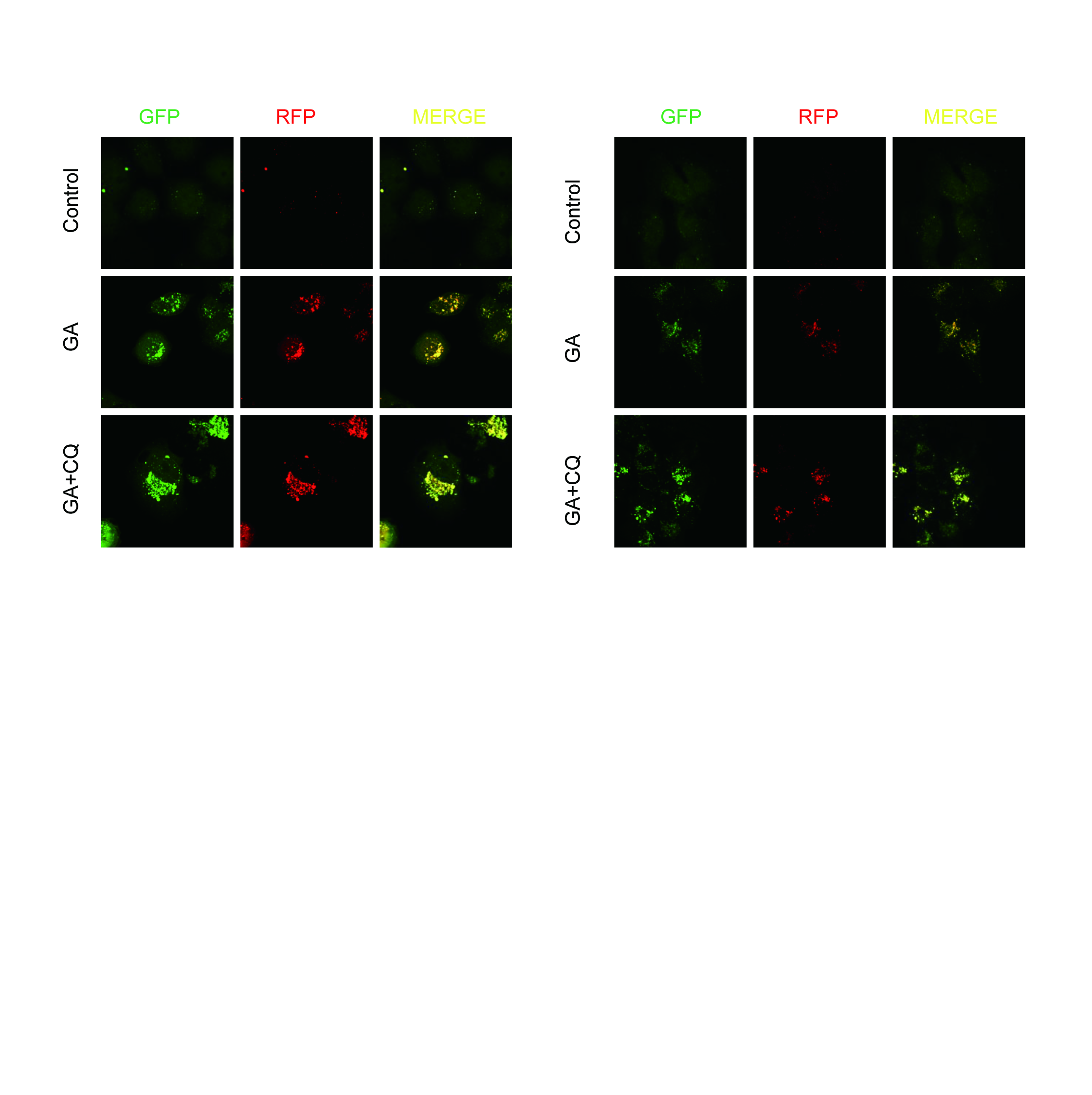
**

**Supplementary Fig. 6**

**
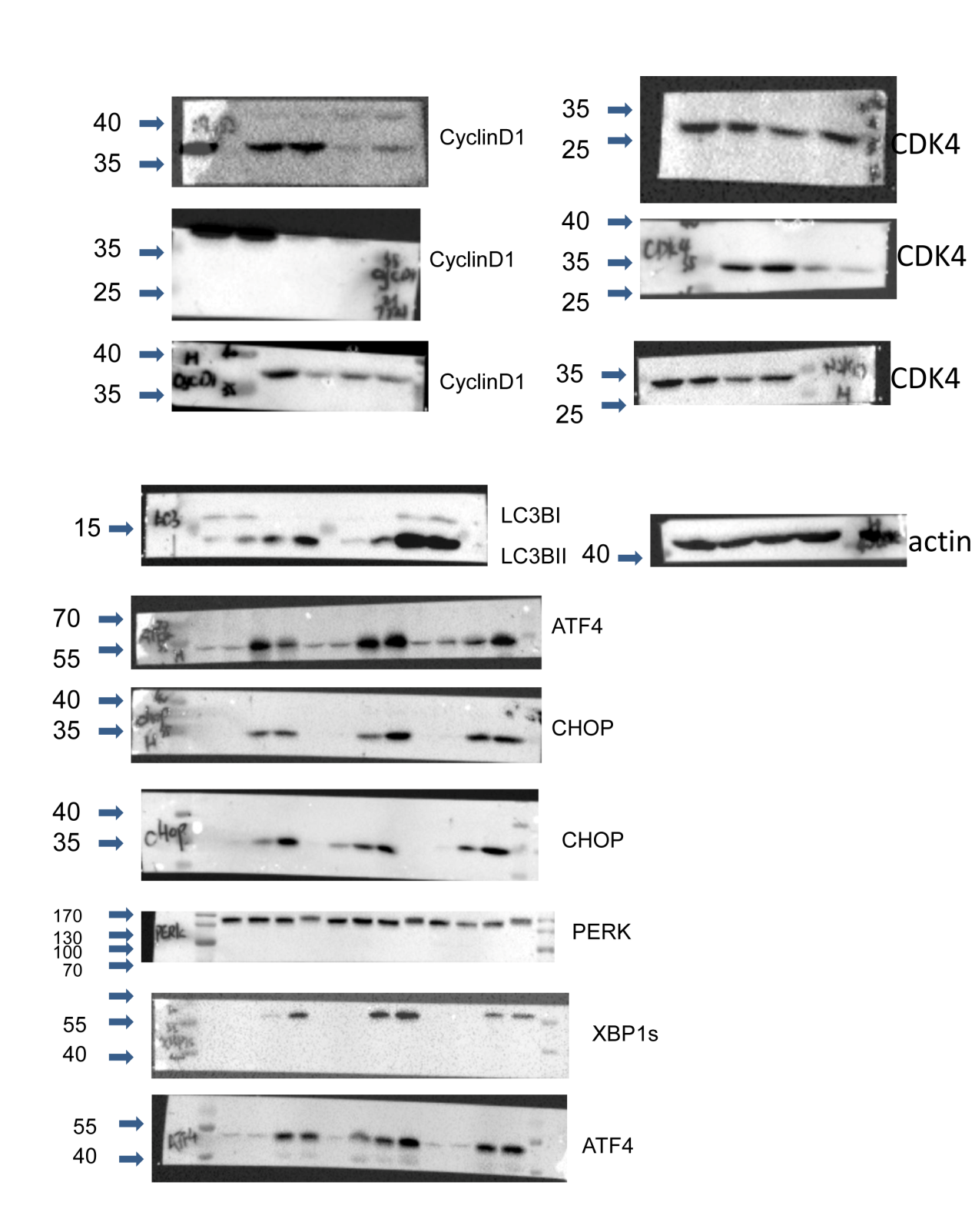
**

**
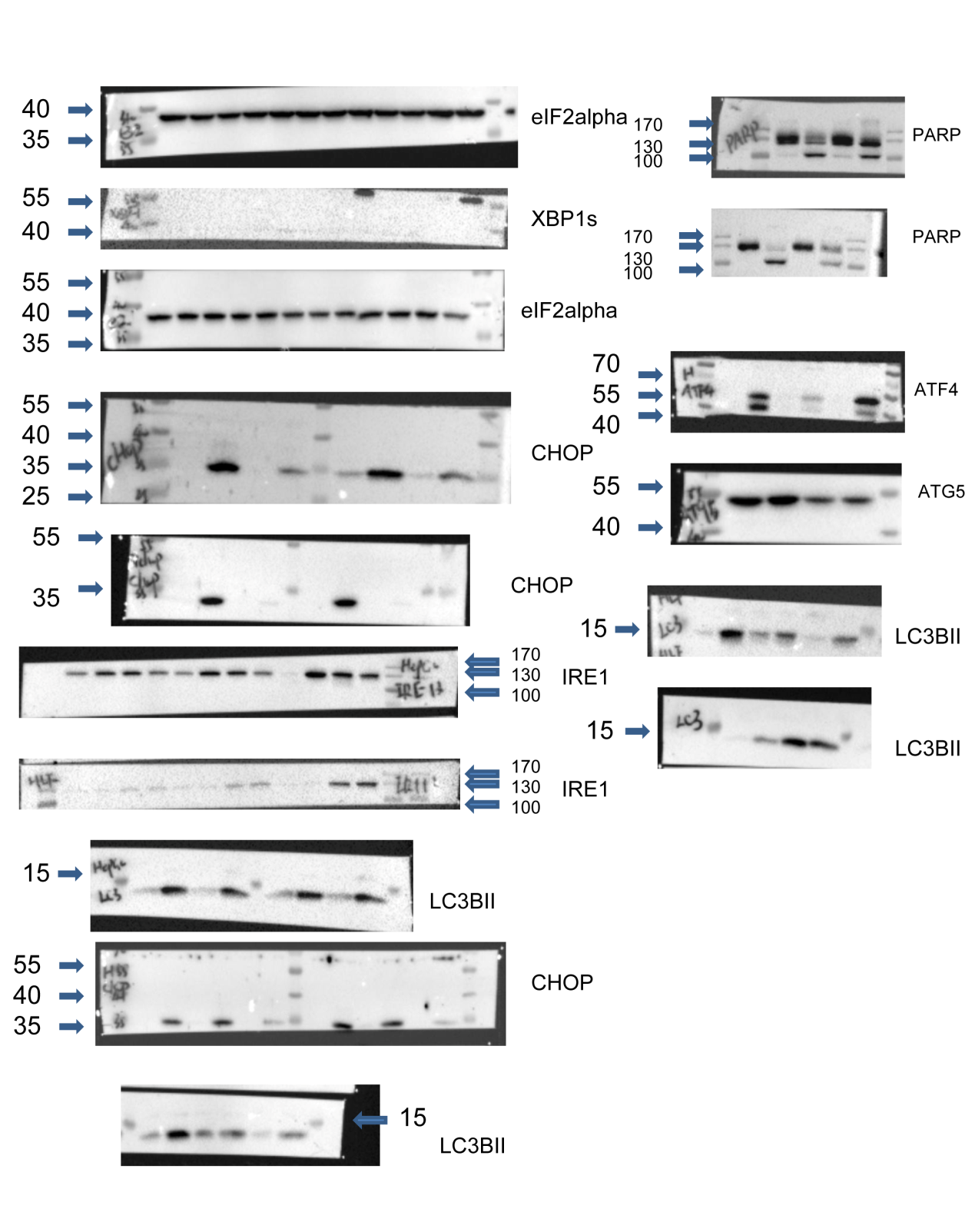
**

**Supplementary Figure 1. GA inhibits the proliferation of HCC cell lines *in vitro* and *in vivo* (a)** The IC50 values of GA in the six HCC cell lines treated at concentrations of 0, 50, 100, 150, 200µM GA for 24h and 48h were calculated. **(b)** 7701 was treated at concentrations of 0, 25, 50, 100, 150, 200uM GA for 24h and 48h, and cell viability was calculated by CCK-8 assay. **(c)** For colony formation assay, HepG2, SMMC-7721 and HLF were treated with indicated concentrations of GA for 36h and then in complete medium for 12 days. Representative images were shown. (**d**) The tumor weight was examined in different groups. (**e**) The body weight of mice in the different groups was shown. (**f**) Immunohistochemical staining of cleaved-caspase3 in control group and GA-treated groups.

**Supplementary Figure 2. GA induces LC3B and CHOP accumulation *in vivo* and ER stress via ROS production (a)** HepG2 and HLF cells were treated various concentrations of GA for 48h, cell cycle analysis was performed by flow cytometry. **(b)** HepG2 cells was subcutaneously injected to the mouse left axillary fossa. Drug administration was started 1 day after tumor implantation. Immunohistochemical staining of ATF4 and CHOP in control group and GA treated groups. Scale bar, 50 µm. **(c)** HepG2 cells was treated with GA (150 µm) with or without Z-VAD-FMK for 24h, cell viability was calculated by CCK8 assay. **(d)** HepG2 and HLF cells were transfected with ATG5 and ATG7 siRNA for 48h, and then treated with GA for another 24h. ATG5 and ATG7 were detected by western blotting. **(e)** HepG2 was pre-treated with or without NAC for 1h following treated with GA for another 24h, CHOP, ATF4 and IRE-1α were detected by western blotting.

**Supplementary Figure 3. Inhibition of autophagy partially relieved ER stress induced by GA. (a)** HepG2 cells were pre-treated with CQ or siRNA for ATG7, then treated with GA for another 24h, P62 was detected by western blotting. **(b-c)** HepG2 cells was treated with GA (150µM) in the presence or absence of Rapamycin (500nm) for 24h. Cell viability was calculated by CCK8 assay and the PARP expression was detected by western blotting. **(d)** HLF was transfected with ATG5 and ATG7 siRNA, or pre-treated with or without CQ for 1h following treated with GA for another 24h, CHOP, IRE-1α, ATG5 and ATG7 were detected by western blotting.

**Supplementary Figure 4** HepG2 and 7721 were treated with various concentrations of GA for 6h, 12h and 24h. The indicated ER stress-related proteins were detected by western blotting. Densitometry graphs of ATF4, CHOP, IRE1α and XBP1s in HLF and 7721 were shown.

**Supplementary Figure 5** HepG2 and HLF cells were transfected with mRFP–GFP-tagged LC3 for 48h, and treated with GA for 24h with or without pretreatment with CQ. Images were taken with a confocal microscope.

**Supplementary Figure 6** We have appended part original and cropped blots in the Supplementary Information file. The corresponding blots can be found in the main diagram of the manuscript. However, we are regret that we are unable to provide the full-length blots of part figures because these blots were previously cropped and we have not obtained the uncropped blot.
